# Effects of Cu (II) on the Growth of *Chlorella vulgaris* and Its Removal Efficiency of Pollutants in Synthetic Piggery Digestate

**DOI:** 10.3390/toxics12010056

**Published:** 2024-01-11

**Authors:** Yaqiong Zeng, Xiaoqing Chen, Jiaming Zhu, Dingbiao Long, Yue Jian, Qiong Tan, Hao Wang

**Affiliations:** 1 Chongqing Academy of Animal Sciences, Chongqing 402460, China; 2 National Center of Technology Innovation for Pigs, Chongqing 402460, China; 3 College of Chemical and Environmental Engineering, Xinjiang Institute of Engineering, Urumqi 830023, China

**Keywords:** *Chlorella vulgaris*, Cu (II), removal efficiency of pollutant, piggery digestate

## Abstract

*C. vulgaris* has a positive effect on the removal of nutrients from pig farm biogas slurry. However, swine wastewater often contains heavy metal ions, such as Cu (II), which may have impacts on the nutrient removal performance of *C. vulgaris*. Additionally, the heavy metal ions in wastewater can be adsorbed by microalgae. In this study, the stress effect of Cu (II) on the growth of *Chlorella vulgaris*, the Cu (II) removal by microalgae, and the effect of different concentrations of Cu (II) on the nutrient removal efficiency of *C. vulgaris* in biogas slurries were explored. The results showed that the microalgae biomass of microalgae on the sixth day of the experiment was the highest in the treatment with a Cu (II) concentration of 0.5 mg/L, which was 30.1% higher than that of the 2.5 mg/L group. *C. vulgaris* had higher removal efficiencies of Cu (II) at a Cu (II) concentration of 0.1~1.5 mg/L. The–OH, C=O, –COOH, and C–O groups on the surface of the algal cells play a significant role in the removal of Cu (II). The removal rates of COD, NH_3_–N, TN, and TP by *C. vulgaris* at a Cu (II) concentration of 0.5 mg/L were the highest, which were 89.0%, 53.7%, 69.6%, and 47.3%, respectively.

## 1. Introduction

Microalgae culturing with piggery digestate can not only realize the resource treatment of piggery wastewater but also produce high value added microalgae biomass, which has broad application prospects [1]. Piggery digestate usually contains high concentrations of ammonia nitrogen (NH_3_–N) and phosphorus chemical oxygen demand (COD), which will cause serious ecological and environmental problems if it is directly discharged into natural water [2,3]. The traditional biogas slurry treatment and utilization methods include land absorption, resource utilization, and biochemical process disposal. Among them, land absorption is simple and low-cost, but it requires a large amount of land, and the continuity of biogas slurry production is often inconsistent with the seasonality of crop irrigation [4,5]. The biochemical treatment includes Anoxic/Oxic, Membrane Bio-Reactor, and other processes. The treated wastewater can meet the discharge standards, but it requires high investment, consumes a lot of energy, and is prone to secondary pollution [6]. In terms of resource utilization, as early as the 1950s, Oswald and Gotaas proposed the concept of using microalgae to treat sewage. Subsequently, many studies coupled microalgae production with wastewater treatment, that is, using sewage to cultivate microalgae [7,8]. The microalgae growth process can remove nitrogen and phosphorus pollutants from wastewater and the wastewater can meet the nutrients needed for microalgae growth, thereby reducing the cost of microalgae cultivation. Using microalgae to treat wastewater can not only produce biomass but also recover nutrients from the wastewater, realizing the resource utilization of wastewater [9,10]. Many studies have shown that microalgae can use high concentrations of nutrients from piggery wastewater. Wen et al. isolated *Chlorella vulgaris* from swine wastewater, and the removal efficiency of the total nitrogen (TN) and total phosphorus (TP) in pig wastewater was 90.51% and 91.54%, respectively [11]. Zhao et al. found that through the co-cultivation of fungi-assisted microalgae, the removal efficiencies of COD, TN, and TP in biogas slurry can reach 92.17 ± 5.28%, 89.83 ± 4.36%, and 90.31 ± 4.69%, respectively [12]. *Chlorella vulgaris JSC-6* was cultivated in swine wastewater for nutrient removal. The results showed that the COD and NH_3_–N removal rates were 60–76% and 40–91%, respectively. Moreover, 3.96 g/L of biomass was produced after 12 days [13]. These studies have shown that microalgae have great potential to remove nutrients from piggery wastewater.

Inorganic forms of copper are often added to diet to satisfy the requirements for the maintenance and growth of pigs. For example, copper sulfate, at a level of 125 to 175 mg/kg, can promote the growth of growing and finishing pigs [14,15]. However, the copper in the feed cannot be completely absorbed by pigs. Approximately 60–70% of the copper is discharged with feces and urine and then enters pig farm wastewater [16,17]. According to the research by Kang et al., the copper content in biogas slurry from large-scale pig farms in China is mostly between 0.03 and 3.50 mg/L, and generally exists in an ionic state [18]. Copper in the form of Cu (II) in wastewater can enter the surrounding water or soil with the discharge of piggery wastewater, seriously threatening human health and the surrounding environment [19,20]. However, copper ions are also trace elements necessary for microalgal growth. Low concentrations of Cu (II) can promote the growth of microalgae. However, excessive Cu (II) concentrations inhibit the growth of microalgae, thereby reducing the removal efficiency of ammonia nitrogen, TP, and COD from piggery wastewater [21]. Therefore, we must consider the influence of Cu (II) on the use of microalgae to treat nutrients in piggery digestate. At present, there are relatively few studies on the mechanisms by which microalgae remove nutrients from biogas slurry wastewater under copper ion stress.

In this study, we investigated the effect of Cu (II) on the growth of *Chlorella vulgaris* and the responses of the microalgae reflected in their physiological and biochemical properties. The Cu (II) removal mechanism by the microalgae and the effect of *Chlorella vulgaris* on the removal efficiency of the nutrients from the biogas slurry under different concentrations of copper ions were also studied. It is expected to provide valuable insights into the treatment of pig farm biogas slurry with microalgae and promote green resource utilization of pig farm wastewater.

## 2. Materials and Methods

### 2.1. Experimental Materials

The *Chlorella vulgaris* used in the experiment was purchased from the Freshwater Algae Culture Collection at the Institute of Hydrobiology. The strain number of the microalgae used was FACHB-8. The chemical reagents, such as the CH_3_COONa, Na_2_CO_3_, NaHCO_3_, Na_3_PO_4_•12H_2_O, (NH_4_)_2_SO_4_, KNO_3_, and NaNO_2_ used to prepare the culture media and adjust the wastewater indicators, were purchased from Shanghai Macklin Bio-Chem Technology Co., Ltd., Shanghai, China.

The synthetic piggery digestate used in the experiment was prepared by adding CH₃COONa (4.0 mg/mL), (NH_4_)_2_SO_4_ (2.6 mg/mL), and K_2_HPO_4_ (0.36 mg/mL) on the basis of the Blue Green (BG11) medium [22]. The methods, indicators, and values for preparing the synthetic wastewater were decided according to the research of Luo et al. [23] and Zhang et al. [24]. The main components of the synthetic wastewater are listed in Table 1.

### 2.2. Culture Conditions

The Cu (II) concentration gradient in the synthetic slurry was adjusted to 0.1, 0.5, 1.5, 2, and 2.5 mg/L by adding CuSO_4_. No additional copper was added in the control group and the Cu (II) concentration in the control group was 0 mg/L (the trace copper elements in the BG11 medium were ignored). The microalgae strain was cultivated in 500 mL flasks with 300 mL of synthetic slurry in a constant temperature light incubator at 25 ± 1 °C under 3400–3500 lux (the daily light/dark cycle was 14:10 h). The inoculum concentration of algae was 0.4 g/L. The flasks were shaken twice daily to keep the light evenly irradiated.

### 2.3. Analytical Determinations

#### 2.3.1. Microalgae Biomass

**Figure 1:**
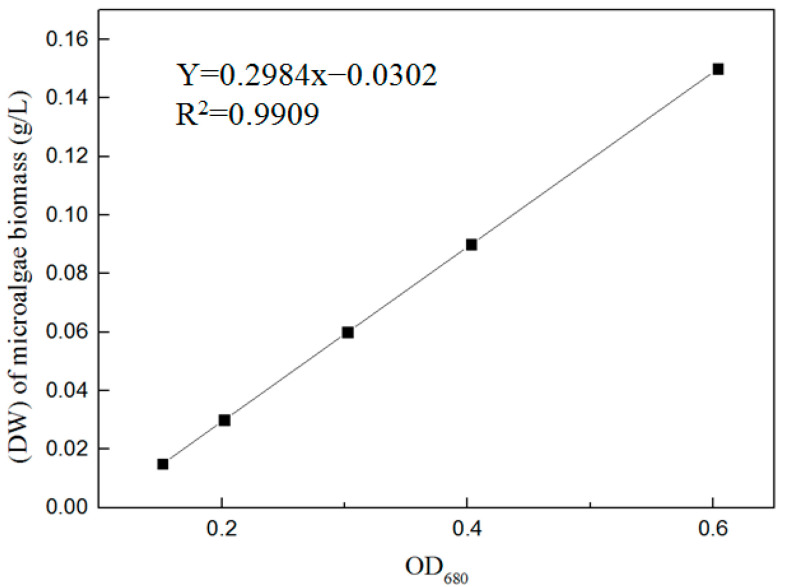
Linear regression between the dry weight of microalgae biomass and optical density.

The drying method [25] was used to obtain a linear relationship between the dry weight (DW) of the microalgae biomass and optical density (OD) at a wavelength of 680 nm in the exponential growth phase (Figure 1). The change in microalgae biomass was analyzed by measuring the OD680 at an interval of 2 d using a UV-3900 spectrophotometer (Hitachi High-Tech Science Co., Ltd., Tokyo, Japan). The DW of the microalgae biomass was calculated according to Equation (1).

Y = 0.2984x − 0.0302, R^2^ = 0.9909 (1)

where Y is the dry weight (DW) of the microalgae biomass (g/L) and x is the OD680.

#### 2.3.2. COD, NH_3_–N, TN, TP

On the eighth day of the experiment, 10 mL of the culture was centrifuged at 10,000× *g* rpm for 5 min at 4 °C. The supernatant was then extracted, and the COD, NH_3_–N, TN and TP were measured using a Hach water quality analyzer [DR6000, Hach Water Quality Analysis Instrument (Shanghai) Co., Ltd., Shanghai, China].

#### 2.3.3. Malondialdehyde (MDA) and Protein

The algal culture (50 mL) was centrifuged at 9000× *g* rpm for 10 min. The precipitate was suspended in 5 mL of phosphate buffer (0.05 M, pH 7.2), which was formulated with 0.05 mol/L NaH_2_PO_4_ and 0.05 mol/L Na_2_HPO_4_ and refrigerated until assayed [26]. The content of MDA and protein was measured using assay kits (Nanjing Mofan Biotechnology Co., Ltd., Nanjing, China). When measuring the MDA content, we added the sample and reagents to the centrifuge tube according to the kit operation sheet, mixed it well, reacted it in a boiling water bath for 20 min, cooled it quickly, and centrifuged it at 4000× *g* rpm/min at room temperature for 10 min. We then took the supernatant and measured the absorbance value at 532 nm, and then used the straight line fitting method to calculate the MDA content. The protein content was determined according to the Coomassie brilliant blue method [27].

#### 2.3.4. Copper Removal Efficiency and Mechanism

The concentration of Cu (II) was measured at 2-day intervals using an inductively coupled plasma spectrometer (Optima 8000, Perkin Elmer Instruments Ltd., Shelton, CT, USA) with 3 replicates per treatment. The changes in Cu (II) concentration with culture time were analyzed and the copper removal efficiency of the microalgae was calculated.

On the eighth day of the experiment, the algae liquid was centrifuged at 3000× *g* rpm for 10 min, and the sedimentation of soybean-sized algae cells was collected at the bottom of a 1.5 mL centrifugal tube. Then, the pre-cold glutaraldehyde fixing liquid was slowly added to the centrifugal tube along the wall, and the sample was stored in a refrigerator at 4 °C until testing. Scanning electron microscopy (SEM: Hitachi S-4800, Hitachi Manufacturing Co., Ltd., Tokyo, Japan) was used to observe the surface shape of the microalgae. In order to analyze the effect of the Cu (II) concentration on the surface morphology of the algal cells, in the SEM analysis, we selected the Cu (II) treatment group with a good microalgae growth status and the excessive Cu (II) concentration treatment group (2.5 mg/L) for comparison and observation. The functional groups of the microalgae samples were investigated using Fourier-transform infrared spectroscopy (FTIR, Thermo Scientific Nicolet 6700, Thermo Fisher Scientific, Waltham, MA, USA) with the spectral wave numbers ranging from 4000 to 500 cm^−1^. The surface composition and chemical states of the samples were investigated using XPS (250XI ESCA, Thermo Fisher Scientific, Waltham, MA, USA) equipped with a Mg Kα X-ray source. In the FTIR and XPS analysis, the control group and the Cu (II) treatment group with a better microalgae status were selected for comparative analysis.

### 2.4. Statistical Analysis

All the experiments were performed in triplicate, and the results were presented as mean ± SD (standard deviation) and charted using OriginPro 9.0. After testing the experimental data for normality and homogeneity of variance, IBM SPSS 20 was used to perform one-way analysis of variance and LSD multiple comparisons. *p* < 0.05 indicates a significant difference.

## 3. Results and Discussion

### 3.1. Microalgae Biomass Analysis

**Figure 2:**
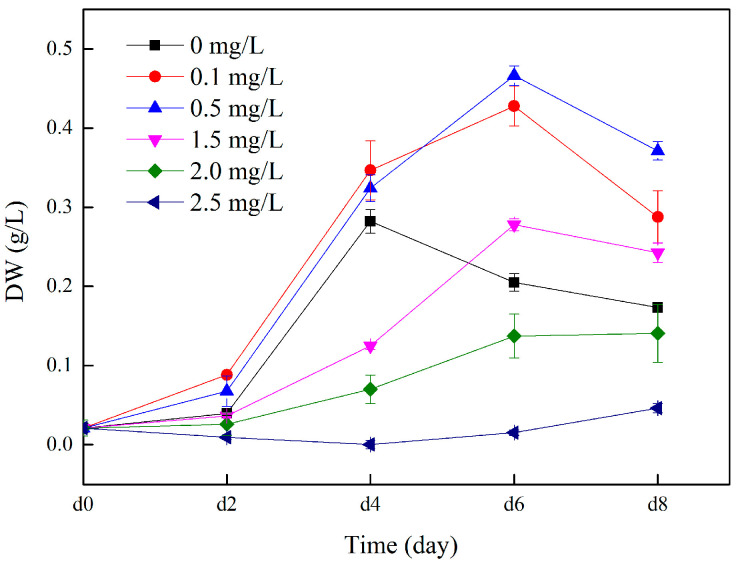
Dynamic changes in microalgae biomass with various concentrations of Cu (II) in swine wastewater.

The effect of Cu (II) on *C. vulgaris* growth is shown in Figure 2. At the beginning of the experiment, the biomass of the microalgae in each treatment group increased slowly or barely. From the second day, the biomass of microalgae in the treatment groups with Cu (II) concentrations of 0.1, 0.5, and 1.5 increased rapidly, reached the highest on the sixth day, and then gradually decreased. This indicates that *C. vulgaris* may have reached a rapid growth stage on the sixth day in this experiment. However, the microalgae biomass gradually decreased after the sixth day, which may also be due to the lack of nutrient supplementation and insufficient nutrients in the culture medium during the experiment. The biomass of the microalgae in the synthetic piggery digestate without Cu (II) increased rapidly on the second to fourth day of culture, and then gradually decreased. Comparable findings were observed in the study by Xi et al. [28], where a higher growth rate of *Chlorella* was noted in a medium with a low concentration of Cu^2+^ (0.1 and 0.4 mg/L) during the initial 1–3 days of the trial, compared to the control group (0 mg/L). On day 8, when the Cu (II) concentration increased from 0.1 to 2.5 mg/L, the biomass of microalgae decreased from 0.288 g/L to 0.046 g/L. During the entire culture period, the microalgae biomass of the treatments with 2.0 and 2.5 mg/L Cu (II) concentrations showed a very slow growth trend. When the Cu (II) concentration exceeded 2.0 mg/L, the microalgae biomass decreased to 33–52% of the control group. This shows that although copper is an essential trace element for algal cells, if the concentration of Cu (II) is higher than 2.0 mg/L, it will inhibit the growth of microalgae. This was also confirmed by Liu et al. [29]. In the Cu(II) concentrations we formulated, the microalgae biomass in the 0.5 mg/L treatment group reached its peak on the sixth day of the experiment, which was 30.1% higher than that of 2.5 mg/L group (*p* < 0.05). At the same time, the biomass of the microalgae in the Cu (II) concentration 0.1 mg/L treatment groups was second only to the 0.5 mg/L group, which was 27.5% higher than the 2.5 mg/L group (*p* < 0.05). In a study by Li et al., it was found that the DW of *C. vulgaris* had no obvious change at a Cu concentration of 0.2 mg/L, while 0.5 mg/L Cu greatly inhibited the growth of the microalgae [30]. This may be due to differences in the media or algae species.

### 3.2. Effect of Cu (II) on MDA and Protein of Microalgae

**Figure 3:**
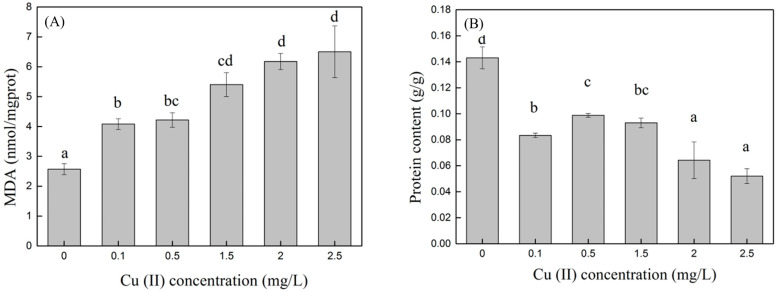
MDA (**A**) and protein (**B**) content in algae cells at different Cu (II) concentrations. Different superscripts for each column indicate significant difference (*p* < 0.05).

When Cu (II) was present in the medium, reactive oxygen species (ROS) were generated in the algal cells due to oxidative stress, and MDA was produced under the influence of ROS. Therefore, the MDA content can indirectly reflect the degree of Cu (II) stress on the algal cells. The higher the MDA content, the greater the damage to the algal cell membranes [21,22,23,24,25,26,27,28,29,30,31,32,33]. The MDA content in algal cells at different Cu (II) concentrations on day eight is shown in Figure 3A. The MDA content in the control group was the lowest, at 2.57 ± 0.18 nmol/mgprot. In the control group, the algal cells only exhibited normal growth and apoptosis, and there was no abnormal membrane lipid peroxidation phenomenon caused by the stimulation of environmental factors; therefore, the MDA content was low. The MDA content gradually increased with an increasing Cu (II) concentration. In synthetic wastewater with Cu (II) concentrations of 0.1, 0.5, 1.5, 2, and 2.5 mg/L, the MDA content in the algal cells was 1.5, 1.6, 2.1, 2.4, and 2.5 times that of the control, respectively, with significant differences (*p* < 0.05). The MDA content of the treatment groups with Cu (II) concentrations of 0.1 and 0.5 mg/L was significantly lower than that of the 1.5, 2, and 2.5 mg/L groups (*p* < 0.05). Combined with Figure 2, at Cu (II) concentrations of 0.1 and 0.5 mg/L, although the MDA content was higher than that of the control group, the microalgae grew well, indicating that at low concentrations of Cu (II), the algae cells would also experience oxidative stress, but it would not affect the growth of the microalgae. However, when the concentration of Cu (II) was higher than 2 mg/L, the algal cells gradually stopped growing and began to die, and the membrane lipid peroxidation reactions in the cell tended to stabilize. Sabatini et al. [34] also showed that an increase in the medium copper concentration induced an increase in the MDA content in *S. vacuolatus* cultivation. The trend of change in MDA was similar to that observed in our experiment.

Heavy metal ions enter microalgae cells and easily combine with other compounds to form metal complexes or chelates, inhibiting various metabolic activities of microalgae, particularly protein synthesis. Therefore, soluble protein content is an important indicator of whether microalgae are under heavy metal stress [35]. As shown in Figure 3B, in this experiment, the protein content of the control group was significantly higher than that of the treatment groups with Cu (II) added (*p* < 0.05). This indicated that *C. vulgaris* was highly sensitive to Cu (II). After adding Cu (II) to the synthetic wastewater, the protein in the microalgae cells was reduced to 36.4–68.5% of the control group. The protein content of the group with a Cu (II) concentration of 0.5 mg/L was 90% higher than that of the group with a Cu (II) concentration of 2.5 mg/L (*p* < 0.05). High concentrations of heavy metals can lead to decreased soluble protein content. The main reason for this is that heavy metals promote the activity of intracellular proteolytic enzymes and strengthen the decomposition of original proteins, leading to the damage of related organelles that synthesize proteins and inhibiting the synthesis of new proteins. The increase in the soluble protein content of the algal cells in the low-concentration heavy metal culture solution was probably a detoxification mechanism for the microalgae to resist the heavy metal toxicity. Heavy metals induce the production of binding proteins, which reduces toxicity [36].

### 3.3. Cu (II) Removal Efficiency and Mechanism Analysis

**Figure 4:**
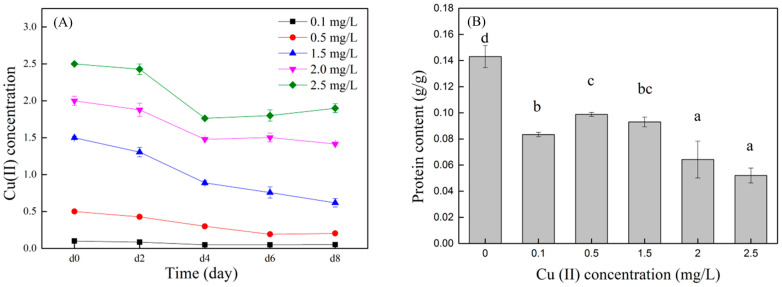
Changes in Cu (II) concentration with culture time (**A**) and Cu (II) removal efficiency by microalgae (**B**). Different superscripts for each column indicate significant difference (*p* < 0.05).

The removal efficiency of copper ions by the *C. vulgaris* is shown in Figure 4. Treatments with lower Cu (II) concentrations (0.1~1.5 mg/L) have higher Cu (II) removal efficiencies than treatments with high Cu (II) concentrations (2 and 2.5 mg/L) (*p* < 0.05). After the microalgae were cultured for eight days, the Cu (II) removal efficiency was the highest in the treatment with a Cu (II) concentration of 0.5 mg/L (59.3%), which was 30.1% (*p* < 0.05) and 35.3% (*p* < 0.05) higher than the treatment groups with Cu (II) concentrations of 2 and 2.5 mg/L, respectively. The treatment groups with Cu (II) concentrations of 0.1 and 1.5 mg/L also had higher Cu (II) removal efficiencies, which were 49.0% and 58.8%, respectively. The removal efficiency of Cu (II) was related to the growth of the microalgae. The lower copper concentration (0.1~1.5 mg/L) in this study can increase the biomass of the microalgae, thereby improving the Cu (II) removal efficiency by the microalgae.

**Figure 5:**
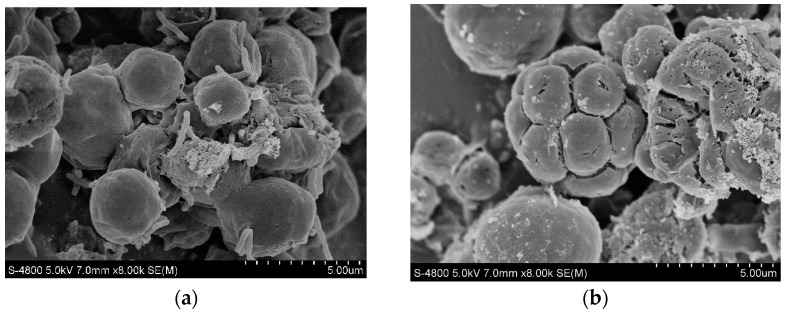
Scanning electron microscope micrograph of *C. vulgaris* at Cu (II) concentrations of 0.5 mg/L (**a**) and 2.5 mg/L (**b**).

SEM images of *C. vulgaris* under appropriate and excessive Cu (II) concentration conditions are shown in Figure 5. *C. vulgaris* had the best growth condition under a Cu (II) concentration of 0.5 mg/L. At this concentration, it can be observed in the SEM image that the cell surface is smooth and round. When the Cu (II) concentration was 2.5 mg/L, wrinkles and cracks appeared on the surface of the algal cells. This indicates that when the Cu (II) concentration is too high, the cells may rupture and become damaged [37], and their growth will be very slow or stop.

**Figure 6:**
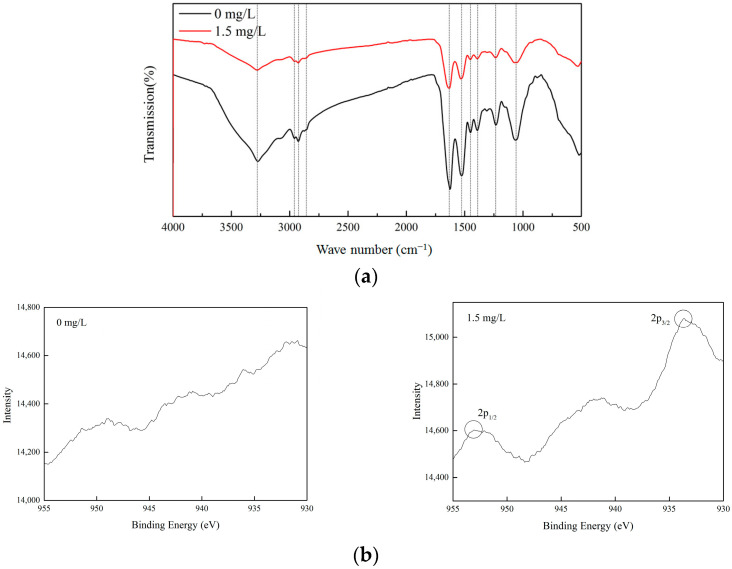
FTIR spectra (**a**) and XPS patterns (**b**) of *C. vulgaris* at Cu (II) concentrations of 0 and 1.5 mg/L.

*C. vulgaris*’s cell walls are mostly composed of polysaccharides, proteins, and lipids, which provide functional groups such as carboxyl, hydroxyl, and amino. These functional groups enable the cell surface to have a negative charge, which shows strong affinity for Cu (II). In the XPS and FTIR analysis, the control (0 mg/L) and the Cu (II) treatment group (1.5 mg/L) with a good microalgae growth status were selected for comparative analysis. The functional groups on the surface of *C. vulgaris* were the main factors affecting its Cu (II) removal performance. The functional groups of microalgae before and after adding Cu (II) were investigated using FTIR. Figure 6a shows the FTIR spectra of the microalgae dried biomass in the spectral range of 400–4000 cm^−1^. After adding Cu (II), the band of the characteristic peaks in the infrared spectrum was basically the same as before the addition, but the intensity of the peaks changed. The stretching vibration characteristic peaks of –OH, C–H, and C=O appeared near 3283 cm^−1^, 2848 cm^−1^ and 2958 cm^−1^, and 1641 cm^−1^, respectively. The characteristic peak near 1533 cm^−1^ may be attributed to the –NH– group [35]. The absorption peak at 1450 cm^−1^ is caused by the stretching vibration of C–OH or –CO–NH– [38]. The characteristic peak at 1400 cm^−1^ was due to the asymmetric stretching vibration of the C–O bond in –COOH. The absorption peaks at 1234 and 1072 cm^−1^ may be attributed to the stretching vibration of the polysaccharide C–O [39]. After adding Cu (II), the characteristic peaks of –OH, C=O, –COOH, and C–O were significantly weakened. It can be seen that –OH, C=O, –COOH, and C–O play a significant role in the removal of Cu (II) by *Chlorella vulgaris*, which is similar to the results reported by Gupta and Rastogi [40].

After adding Cu (II), the state of the copper ions on *C. vulgaris* was characterized using XPS, as shown in Figure 6b. The results showed that no characteristic peaks of copper were observed on the microalgae before adding Cu (II) (0 mg/L). Peaks corresponding to the 2p3/2 and 2p1/2 with a binding energy of 934.5 eV and 954.1 eV, respectively, were found on the microalgae cells after the treatment with Cu (II) (1.5 mg/L) [29]. This indicated the presence of Cu (II) species.

### 3.4. Effect of Cu (II) on the Efficiency of Microalgae in Degrading Nutrients in Wastewater

**Figure 7:**
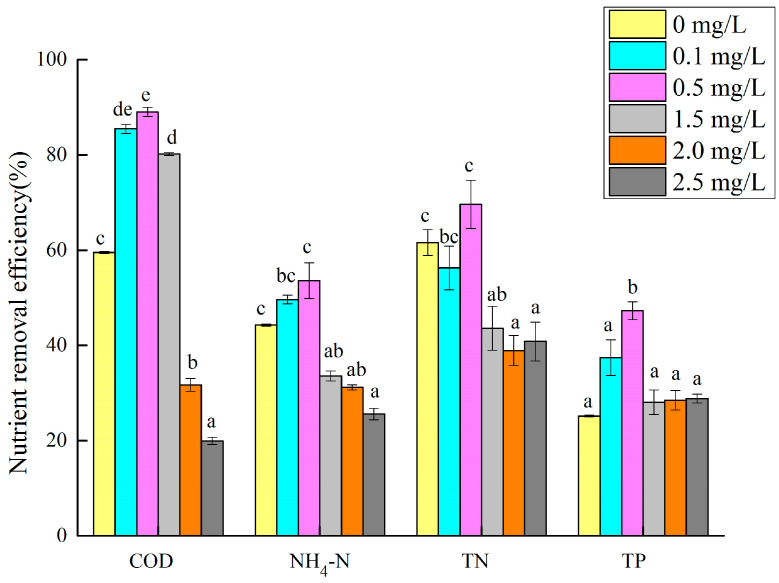
Nutrient removal rate of microalgae cultured in wastewater with different Cu (II) concentrations. Different superscripts for each nutrient indicate significant difference (*p* < 0.05).

The removal efficiency of the microalgae for the nutrients in the synthetic biogas slurry was different with different Cu (II) concentrations. The nutrient removal efficiency of the microalgae with different Cu (II) concentrations is shown in Figure 7. In the treatment group with a Cu (II) concentration of 0.5 mg/L, the removal rates of COD, NH_3_–N, TN, and TP by the microalgae were the highest (89.0%, 53.7%, 69.6%, and 47.3%, respectively), all of which were significantly higher than those of the 1.5, 2, and 2.5 mg/L groups (*p* < 0.05). Combined with Figure 2, at the end of the test, the 0.5 mg/L treatment group had the largest microalgae biomass, and the removal rate of nutrients in the synthetic biogas slurry by the microalgae was also higher. Li, X. et al. found that when the Cu (II) concentration was 0.5 mg/L, the removal rates of NH_3_–N and TP in the digested swine wastewater by *C. vulgaris* were 58.8% and 84.9%, respectively, similar to the results of this study. However, they noted that the microalgae growth was inhibited at all Cu (II) concentrations, possibly because of differences in the incubation time or wastewater composition [41]. When the Cu (II) concentration was between 2.0 and 2.5 mg/L, the nutrient removal rate in the synthetic biogas slurry was the lowest. This may be because the high concentration of Cu (II) has a stress effect on the growth of the microalgae, and the microalgae cannot multiply rapidly, or die. Chen et al. reported that the removal of nitrogen, phosphorus, and ammonia nitrogen from digested swine wastewater was 87.68–89.85%, 92.61–93.68%, and 97.02–97.86% by *Chlorella vulgaris FACHB-31* and *C. vulgaris FACHB-8* [42]. Xu et al. [22] showed that the nutrient removal efficiency of microalgae from a cultivation medium with digested effluent was 74.63% TN and 81.73% TP. Not many studies have focused on the removal of nutrients from wastewater by microalgae under copper ion stress. *C. vulgaris* in this study can adapt to digested swine wastewater with copper ions and conventional nutrient concentrations, and has good removal effects on COD, NH_3_–N, TN, and TP.

## 4. Conclusions

*C. vulgaris* grew well in the synthetic biogas slurry and could remove pollutants from swine wastewater in the presence of Cu (II). The biomass of *C. vulgaris* performed the best with a Cu (II) concentration of 0.5 mg/L and the microalgae had the highest removal efficiency of nutrients in wastewater at this concentration. An excessive Cu (II) concentration will inhibit the growth of the microalgae and the removal efficiency of nutrients from the wastewater. At the same time, excess Cu (II) might suppress the removal of copper by the microalgae. *C. vulgaris* is an economic and environmentally friendly biological resource, which could simultaneously remove nutrients and Cu from livestock wastewater. However, in actual applications, the appropriate algae must be selected according to the characteristics of the wastewater. If necessary, certain pre-treatments of the wastewater should be performed to achieve good treatment effects.

## Figures and Tables

**Table 1:** Basic properties of synthetic piggery digestate.

COD (mg/L)	NH_4_–N (mg/L)	TN (mg/L)	TP (mg/L)	pH
1867.78 ± 63.11	482.86 ± 15.17	749.44 ± 113.47	71.64 ± 3.87	6.33 ± 0.05

Note: Values are presented as mean ± SD. COD = chemical oxygen demand, NH_4_–N = ammonia nitrogen, TN = total nitrogen, TP = total phosphorus.

## Data Availability

All the data generated or analyzed during this study are included in this article.
